# Systematic review of autosomal recessive ataxias and proposal for a classification

**DOI:** 10.1186/s40673-017-0061-y

**Published:** 2017-02-23

**Authors:** Marie Beaudin, Christopher J. Klein, Guy A. Rouleau, Nicolas Dupré

**Affiliations:** 1 Faculty of Medicine, Université Laval, Quebec city, QC G1V 0A6 Canada; 2 Department of Neurology, Mayo Clinic, Rochester, MN 55905 USA; 3 Department of Neurology and Neurosurgery, McGill University, Montreal, QC H3A 1A4 Canada; 4 Department of Neurological Sciences, CHU de Quebec - Université Laval, 1401 18th street, Québec City, QC G1J 1Z4 Canada

**Keywords:** Cerebellar ataxia, Spinocerebellar degenerations, Recessive, Genetics, Classification

## Abstract

**Background:**

The classification of autosomal recessive ataxias represents a significant challenge because of high genetic heterogeneity and complex phenotypes. We conducted a comprehensive systematic review of the literature to examine all recessive ataxias in order to propose a new classification and properly circumscribe this field as new technologies are emerging for comprehensive targeted gene testing.

**Methods:**

We searched Pubmed and Embase to identify original articles on recessive forms of ataxia in humans for which a causative gene had been identified. Reference lists and public databases, including OMIM and GeneReviews, were also reviewed. We evaluated the clinical descriptions to determine if ataxia was a core feature of the phenotype and assessed the available evidence on the genotype-phenotype association. Included disorders were classified as primary recessive ataxias, as other complex movement or multisystem disorders with prominent ataxia, or as disorders that may occasionally present with ataxia.

**Results:**

After removal of duplicates, 2354 references were reviewed and assessed for inclusion. A total of 130 articles were completely reviewed and included in this qualitative analysis. The proposed new list of autosomal recessive ataxias includes 45 gene-defined disorders for which ataxia is a core presenting feature. We propose a clinical algorithm based on the associated symptoms.

**Conclusion:**

We present a new classification for autosomal recessive ataxias that brings awareness to their complex phenotypes while providing a unified categorization of this group of disorders. This review should assist in the development of a consensus nomenclature useful in both clinical and research applications.

**Electronic supplementary material:**

The online version of this article (doi:10.1186/s40673-017-0061-y) contains supplementary material, which is available to authorized users.

## Background

The classification of the hereditary ataxias has represented a challenge for decades due to the large heterogeneity of clinical presentations and the important overlap between different pathologies [1]. The first to propose a global classification for this group of disorders was Greenfield in 1954, whose classification was based on pathoanatomical findings [2]. This was followed by Harding’s classification in 1983, which regrouped the ataxias according to age of onset, as a proxy for mode of inheritance, and clinical findings [3]. Although this clinical classification had merit, it quickly became overshadowed by a nomenclature based on gene discoveries within each specific type of ataxia starting with *ATXN1* in Spinocerebellar ataxia 1 in 1993 [4] and *FXN* in Friedreich ataxia [5]. Since then, over 40 genes have been discovered in the dominant ataxias and as many in recessive ataxias [6].

One of the main challenges in the study of recessive ataxias is the difficulty to properly circumscribe which disorders belong to the field of hereditary ataxias and which belong to other disease categories. Indeed, ataxia is a cardinal symptom in cerebellar disorders, but may also be a presenting symptom of hereditary spastic paraplegias, hereditary polyneuropathies, neurodevelopmental disorders, and mitochondrial diseases, for example. Concurrently, recessive ataxias often manifest with complex phenotypes, even more so than their dominant counterparts, and may present diverse associated features including neuropathy, pyramidal and extrapyramidal involvement, oculomotor abnormalities, cognitive involvement, seizures, retinopathy, hypogonadism, and many others. This explains the high variability in the list of included disorders in recent literature reviews on recessive ataxias [7, 8].

Nevertheless, the advent of next generation sequencing techniques requires to properly determine which disorders belong to each disease category in order to design thoughtful targeted panels and facilitate the interpretation of whole exome and whole genome sequencing data. Indeed, targeted panel sequencing is a highly effective method for the diagnosis of neurological disorders, but it requires insightful categorization of disease phenotypes to respond to the specific needs of clinicians [9, 10]. Similarly, the interpretation of unknown variants in the analysis of whole exome or whole genome sequencing data poses a significant challenge for clinicians who must determine if the gene is associated with the suspected disease category and if the phenotype correlates with what has previously been described. As next generation sequencing techniques become increasingly available and the ability to detect DNA repeat expansion diseases improves [11], the proper classification of diseases will represent a useful tool in the interpretation of test results. Hence, this calls for a systematic effort to review recessive diseases in which ataxia is a prominent feature in order for experts in the field to collectively determine which disorders should be included in a recessive ataxia classification.

Therefore, the purpose of this article is to review the literature on recessive diseases presenting with ataxia in order to present a new classification. The goal is to bring together experts for the development of a much-needed consensus that fulfills research and clinical needs.

## Methods

We conducted a systematic review to identify articles relevant to the classification of autosomal recessive ataxias. We searched Pubmed and Embase from inception to September 2016 in order to identify original articles on disorders presenting with ataxia. The search strategy was large and targeted both recessive and sporadic ataxias, since recessive inheritance may appear sporadic in certain circumstances (full search strategy is provided in Additional file 1). We also reviewed reference lists of relevant articles and public databases including OMIM and GeneReviews to identify other relevant articles.

We reviewed the titles and abstracts of all identified references to select original articles on recessive forms of ataxia in humans for which a causative gene was identified. We evaluated the articles from a clinical perspective to determine if cerebellar ataxia was a prominent feature in the reported patients or rather a secondary finding in other movement or multisystem diseases. Diseases reporting only on cerebellar atrophy or cerebellar malformations without any clinical consequence were not included. For each listed disorder, we reviewed the evidence for a genotype-phenotype association using the US National Human Genome Research Institute guidelines [12]. Major considerations included the exclusion of previously described genes, the number of unrelated individuals described with similar genotype-phenotype correlations, the evidence of segregation with the disease, the absence of the variant in large control cohorts, and the presence of biochemical or animal-model functional validation. For the primary ataxias, we identified two relevant references from different research groups when possible. All relevant articles were fully reviewed to be included in this classification of recessive ataxias.

Identified disorders were classified in three categories: the first included the primary autosomal recessive ataxias, the second included other movement or multisystem recessive diseases that have prominent ataxia, and the final group was composed of recessive disorders that may occasionally present with ataxia, but where ataxia is a secondary feature.

We also developed a clinical algorithm for the primary recessive ataxias based on the most frequent phenotype and cardinal symptoms associated with each disorder. The objective of this algorithm is to rapidly summarize the main discriminatory features between different ataxias to serve in a clinical setting, but also as a pedagogical and research tool.

## Results

**Fig. 1:**
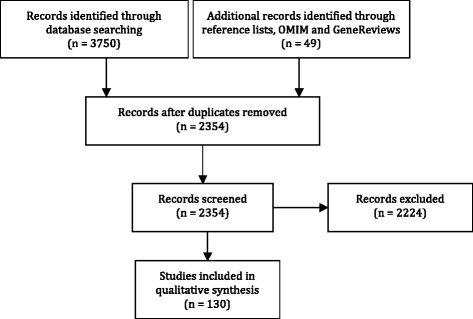
Flow diagram

3750 references were identified through the literature search in Pubmed and Embase, and 49 additional references were identified through reference lists or public databases. After removal of duplicates, 2354 references were reviewed on the basis of title and abstract. Finally, 130 articles were selected on the basis of the aforementioned criteria and completely reviewed to be included in this qualitative analysis (Fig. 1).

The proposed new list of autosomal recessive ataxias is presented in Table 1 in chronological order of gene discovery. The disorders included in this list were evaluated as having a relatively predominant cerebellar involvement compared to the involvement of other neurologic and non-neurologic systems. Table 2 presents the other complex motor or multisystem disorders that have prominent ataxia. Finally, Table 3 presents disorders that may occasionally present with ataxia, but where ataxia is a secondary feature. Certain decisions were made in the elaboration of this classification. Notably, abetalipoproteinemia (ABL) and Refsum disease were not included in the list of primary recessive ataxias, but rather in the list of complex disorders that have prominent ataxia. Indeed, despite their important Friedreich-like neurological picture, these disorders are primary lipid metabolism disorders with multisystem involvement. Moreover, ataxic disorders that are allelic to other movement disorders, especially spinocerebellar ataxias and hereditary spastic paraplegias, were assigned to the second category to avoid any confusion with the primary recessive ataxias. The MARS2-linked autosomal recessive ataxia with leukoencephalopathy (ARSAL/SPAX3) was not included because the genetic evidence was deemed insufficient [13]. Finally, some disorders described only in single families were included, despite this being a factor for weaker genetic evidence, if other major considerations were met; this was indicated in the list.

**Table 1:** Proposed new list of autosomal recessive ataxias

Disorder	Gene	OMIM	Additional clinical features and neuroimaging findings	Relevant references
CTX	*CYP27A1*	213700	Dementia, paresis, tendon xanthomas, atherosclerosis, cataracts, elevated cholestanol level, childhood onset, variable cerebellar atrophy, cerebellar or cerebral leukodystrophy	[17, 18]
AVED	*TTPA*	277460	Retinitis pigmentosa, head titubation, low serum vitamin E, teenage onset, spinal cord atrophy, absence of cerebellar atrophy	[19, 20]
AT	*ATM*	208900	Telangiectasias, oculomotor apraxia, photosensitivity, immunodeficiency, predisposition for cancer, elevation of α-foetoprotein, infantile onset, cerebellar atrophy	[21, 22]
FRDA	*FXN*	229300	Bilateral Babinski sign, square-wave jerks, scoliosis, hypertrophic cardiomyopathy, sensory involvement, teenage onset, spinal cord atrophy, absence of cerebellar atrophy	[5, 23]
ATLD	*MRE11*	604391	Oculomotor apraxia, childhood onset, cerebellar atrophy	[24, 25]
ARSACS	*SACS*	270550	Spastic paraparesis, retinal striation, pes cavus, infantile or childhood onset, anterior superior cerebellar atrophy, occasional T2-weighted linear hypointensities in pons	[26, 27]
AOA1/EAOH	*APTX*	208920	Oculomotor apraxia, cognitive impairment, hypoalbuminemia, hypercholesterolemia, childhood onset, cerebellar atrophy	[28, 29]
SCAN1	*TDP1*	607250	Peripheral axonal sensorimotor neuropathy, distal muscular atrophy, hypercholesterolemia, teenage onset, cerebellar atrophy	[30, 31]
Cayman ataxia	*ATCAY*	601238	Psychomotor retardation, hypotonia, strabism, neonatal onset, cerebellar hypoplasia	[32, 33]
SANDO or MIRAS/SCAE	*POLG1*	607459	In SANDO, sensory ataxia, ophtalmoparesis, myoclonus, ptosis, adult onset, variable cerebellar atrophy, cerebellar white matter lesions, strokelike lesions. In MIRAS, cerebellar and sensitive ataxia, epilepsy, migraine, myoclonus, childhood or teenage onset, signal abnormalities in cerebellum and thalamus	[34, 35]
AOA2	*SETX*	606002	Polyneuropathy, pyramidal signs, oculomotor apraxia, head tremor, chorea, dystonia, elevation of α-foetoprotein, teenage onset, cerebellar atrophy	[36, 37]
CAMRQ1, DES	*VLDLR*	224050	Non-progressive cerebellar ataxia, mental retardation, hypotonia, strabismus, occasional quadripedal gait, congenital onset, inferior cerebellar hypoplasia, cortical gyral simplification	[38, 39]
IOSCA/MTDPS7 (Allelic to PEOA3)	*C10orf2*	271245	Athetosis, hypotonia, optic atrophy, ophtalmoplegia, hearing loss, epilepsy, hypogonadism, liver involvement, infantile onset, moderate atrophy of brainstem and cerebellum with advancing disease	[40, 41]
MSS	*SIL1*	248800	Cataracts, mental retardation, myopathy, short stature, childhood onset, cerebellar atrophy	[42, 43]
DCMA/MGCA5	*DNAJC19*	610198	Dilated cardiomyopathy, non-progressive cerebellar ataxia, mental retardation, testicular dysgenesis, anemia, increased urinary 3-methylglutaconic acid, infantile onset	[44, 45]
ARCA1	*SYNE1*	610743	Pure cerebellar ataxia, cognitive impairment, occasional pyramidal signs, late onset, cerebellar atrophy	[46, 47]
ARCA2	*ADCK3* (*CABC1*)	612016	Exercise intolerance, epilepsy, myoclonus, cognitive impairment, childhood onset, cerebellar atrophy, occasional strokelike cerebral lesions	[48, 49]
SeSAME syndrome	*KCNJ10*	612780	Epilepsy, sensorineural deafness, mental retardation, tubulopathy and electrolyte imbalance, infantile onset, absence of cerebellar atrophy	[50, 51]
CAMRQ3	*CA8*	613227	Mild mental retardation, occasional quadrupedal gait, congenital onset, cerebellar atrophy, white matter abnormalities	[52, 53]
Salih ataxia/SCAR15 (1 family)	KIAA0226	615705	Epilepsy, mental retardation, childhood onset, absence of cerebellar atrophy	[54, 55]
PHARC	*ABHD12*	612674	Sensorimotor neuropathy, cataract, hearing loss, retinitis pigmentosa, teenage onset, variable cerebellar atrophy	[56, 57]
SPAX4 (1 family)	*MTPAP*	613672	Spastic paraparesis, optic atrophy, cognitive involvement, infantile onset	[58, 59]
ARCA3	*ANO10*	613728	Cognitive impairment, downbeat nystagmus, teenage or adult onset, cerebellar atrophy	[60, 61]
SCAR11 (1 family)	*SYT14*	614229	Psychomotor retardation, late onset, cerebellar atrophy	[62]
CAMRQ2	*WDR81*	610185	Occasional quadrupedal gait, cognitive impairment, congenital onset, hypoplasia of cerebellum and corpus callosum	[63, 64]
AOA3 (1 family)	*PIK3R5*	615217	Oculomotor apraxia, sensorimotor involvement, teenage onset, cerebellar atrophy	[65]
SCAR13	*GRM1*	614831	Cognitive impairment, mild pyramidal signs, short stature, seizures, congenital onset, cerebellar atrophy	[66, 67]
CAMRQ4 (1 family)	*ATP8A2*	615268	Cognitive impairment, occasional quadrupedal gait, congenital onset, cerebellar and cerebral atrophy	[68]
SCAR7 (Allelic to CLN2)	*TPP1*	609270	Pyramidal signs, posterior column involvement, tremor, childhood onset, atrophy of the cerebellum and pons	[69, 70]
Ataxia and hypogonadotropism	*RNF216*	212840	Hypogonadotropic hypogonadism, dementia, occasional chorea, childhood to young adult onset, cerebellar and cerebral atrophy	[71, 72]
SCAR18	*GRID2*	616204	Tonic upgaze, psychomotor retardation, retinal dystrophy, infantile onset, cerebellar atrophy	[73, 74]
SCAR16	*STUB1*	615768	Pyramidal signs, neuropathy, occasional hypogonadism, variable age at onset, cerebellar atrophy	[75, 76]
SCAR12	*WWOX*	614322	Tonic-clonic epilepsy, mental retardation, spasticity, neonatal to childhood onset, variable cerebellar or cerebral atrophy	[77, 78]
ATLD2 (1 family)	*PCNA*	615919	Telangiectasias, sensorineural hearing loss, photosensitivity, cognitive impairment, short stature, childhood onset, cerebellar atrophy	[79]
SCAR20	*SNX14*	616354	Mental retardation, sensorineural hearing loss, macrocephaly, dysmorphism, infantile onset, cerebellar atrophy	[80, 81]
SCAR17	*CWF19L1*	616127	Mental retardation, congenital onset, cerebellar hypoplasia	[82, 83]
ACPHD (1 family)	*DNAJC3*	616192	Diabetes mellitus, UMN signs, demyelinating neuropathy, sensorineural hearing loss, childhood to adult onset, generalized supra- and infratentorial atrophy	[84]
LIKNS/SCAR19 (1 family)	*SLC9A1*	616291	Sensorineural hearing loss, childhood onset, variable vermian atrophy	[85]
AOA4 (Allelic to MCSZ)	*PNKP*	616267	Dystonia, oculomotor apraxia, polyneuropathy, cognitive impairment, childhood onset, cerebellar atrophy	[86, 87]
SCAR2	*PMPCA*	213200	Non-progressive cerebellar ataxia, cognitive impairment, pyramidal signs, short stature, congenital or infantile onset, cerebellar atrophy	[88, 89]
SCAR21	*SCYL1*	616719	Liver failure, peripheral neuropathy, mild cognitive impairment, childhood onset, cerebellar vermis atrophy, thinning of optic nerve	[90]
SCAR22 (1 family)	*VWA3B*	616948	Cognitive impairment, pyramidal signs, adult onset, cerebellar atrophy and thin corpus callosum	[91]
SCAR23 (1 family)	*TDP2*	616949	Tonic seizures, cognitive impairment, dysmorphism, childhood onset	[92]
SCAR24 (1 family)	*UBA5*	617133	Cataracts, peripheral neuropathy, childhood onset, cerebellar atrophy	[93]
Cerebellar ataxia with developmental delay (1 family)	*THG1L*	-	Psychomotor retardation, pyramidal signs, childhood onset, vermis hypoplasia	[94]

*ACPHD* Ataxia, combined cerebellar and peripheral, with hearing loss and diabetes mellitus, *AOA* ataxia with oculomotor apraxia, *ARCA* autosomal recessive cerebellar ataxia, *ARSACS* autosomal recessive spastic ataxia of Charlevoix-Saguenay, *AT* ataxia-telangiectasia, *ATLD* ataxia-telangiectasia-like disorder, *AVED* ataxia with vitamin E deficiency, *CA* Cayman ataxia, *CAMOS* cerebellar ataxia mental retardation optic atrophy and skin abnormalities, *CAMRQ* cerebellar ataxia mental retardation with or without quadrupedal locomotion, *DCMA* Dilated cardiomyopathy with ataxia, *DES* Desequilibrium syndrome, *EAOH* early-onset ataxia with oculomotor apraxia and hypoalbuminemia, *FRDA* Friedreich ataxia, *IOSCA* infantile onset spinocerebellar ataxia, *LIKNS* Lichtenstein-Knorr syndrome, *MGCA5* 3-methyglutaconic aciduria type 5, *MIRAS* mitochondrial recessive ataxia syndrome, *MCSZ* Microchephaly seizures developmental delay, *MSS* Marinesco-Sjogren syndrome, MTDPS7 mitochondrial DNA depletion syndrome 7, *PEOA3* progressive external ophthalmoplegia with mitochondrial DNA deletions, autosomal dominant 3, *PHARC* polyneuropathy hearing loss ataxia retinitis pigmentosa and cataract, *SANDO* sensory ataxic neuropathy with dysarthria and ophthalmoparesis, *SCAE* spinocerebellar ataxia with epilepsy, *SCAN1* spinocerebellar ataxia with axonal neuropathy 1, *SCAR* Spinocerebellar ataxia, autosomal recessive, *SeSAME* Seizures sensorineural deafness ataxia mental retardation and electrolyte imbalance, *SPAX* spastic ataxia, *UMN* upper motor neuron

**Table 2:** Other complex movement or multisystem recessive disorders that have prominent ataxia

Disorder	Gene	OMIM	Clinical features and imaging findings	Comment	References
Abetalipoproteinemia	*MTTP*	200100	Fat malabsorption symptoms, hypocholesterolemia, hypotriglyceridemia, acanthocytosis, Friedreich-like ataxia, neonatal onset, absence of cerebellar atrophy	Multisystem	[95]
Nieman Pick type C	*NPC1*	257220	Vertical supranuclear ophtalmoplegia, ataxia, splenomegaly, childhood to adult onset, variable cerebellar or cerebral atrophy	Multisystem	[96, 97]
	*NPC2*	607625			
Refsum disease	*PAHX*	266500	Retinitis pigmentosa, polyneuropathy, ataxia, increased CSF protein, anosmia, deafness, ichtyosis, teenage onset, elevated serum phytanic acid, absence of cerebellar atrophy	Multisystem	[98, 99]
Late-onset GM2 gangliosidosis (Tay-Sachs, Sandhoff)	*HEXA HEXB*	272800 268800	Ataxia, dysarthria, intellectual impairment, extrapyramidal signs, adult onset, cerebellar atrophy	Lysosomal storage disease	[100–102]
SPARCA1	*SPTBN2*	615386	Ataxia, cognitive impairment, eye-movement abnormalities, early childhood onset, cerebellar atrophy	Allelic to SCA5	[9, 103]
SPAX5	*AFG3L2*	614487	Ataxia, spasticity, oculomotor apraxia, myoclonic epilepsy, neuropathy, dystonia, optic atrophy, childhood onset, cerebellar atrophy	Allelic to SCA28	[104, 105]
Boucher-Neuhauser/Gordon Holmes syndrome	*PNPLA6*	215470	Ataxia, hypogonadotropic hypogonadism, chorioretinal dystrophy or brisk reflexes, childhood onset, atrophy of cerebellum and pons	Allelic to HSP39	[106, 107]
Gillespie syndrome	*ITPR1*	206700	Non-progressive cerebellar ataxia, iris hypoplasia, cognitive impairment, neonatal onset, progressive cerebellar atrophy	Allelic to SCA15/29	[108]
SPAX2/SPG58	*KIF1C*	611302	Spastic paraparesis, cerebellar ataxia, childhood or teenage onset, white matter changes in the internal capsule	Spasticity predominant	[109, 110]
SPG7	*SPG7*	607259	Spasticity, pyramidal signs, cerebellar signs, optic neuropathy, ptosis, teenage or adult onset, cerebellar atrophy	HSP	[111, 112]
SPG5	*CYP7B1*	270800	Spasticity, cerebellar and sensory ataxia, childhood or teenage onset, white matter lesions	HSP	[113, 114]
SPG11	*KIAA1840*	604360	Spasticity, ataxia, cognitive impairment, sensorimotor neuropathy, childhood or teenage onset, thin corpus callosum, signal abnormalities in cervical cord	HSP	[115, 116]
SPG46	*GBA2*	614409	Cerebellar ataxia, spastic dysarthria, mild cognitive impairment, hearing loss, cataracts, childhood onset, cerebellar and cerebral atrophy, thin corpus callosum	HSP	[117, 118]
Congenital disorders of glycosylation type 1A	*PMM2*	212065	Psychomotor retardation, axial hypotonia, abnormal eye movements, peripheral neuropathy, congenital onset, cerebellar hypoplasia	Neonatal onset, complex syndrome	[119, 120]
LBSL	*DARS2*	611105	Cerebellar ataxia, tremor, spasticity, dorsal column dysfunction, axonal neuropathy, childhood to adult onset, signal abnormalities in cerebral white matter and specific brainstem and spinal cord tracts	Leukoencephalopathy	[121, 122]
Mitochondrial complex IV deficiency	*COX20*	220110	Cerebellar ataxia, dystonia, sensory axonal neuropathy, variable, childhood or teenage onset, cerebellar atrophy	Dystonia predominant	[123]
Aceruloplas-minemia	*CP*	604290	Diabetes, dementia, movement disorder, cerebellar ataxia, retinal degeneration, late onset, decreased signal intensity in thalamus, basal ganglia and dentate nucleus	Metabolic disorder	[124]
Neurodegeneration with brain iron accumulation 2A and 2B	*PLA2G6*	256600	Cerebellar ataxia, psychomotor retardation, psychiatric features, axonal sensorimotor neuropathy, infantile or teenage onset, cerebellar atrophy and variable iron accumulation in globus pallidus	Neurodegeneration with brain iron accumulation	[125, 126]
Poretti-Botshauser syndrome	*LAMA1*	615960	Nonprogressive ataxia, oculomotor ataxia, psychomotor retardation, early childhood onset, cerebellar dysplasia with cysts	Dystroglycanopathy	[127]
Posterior column ataxia with retinitis pigmentosa	*FLVCR1*	609033	Posterior column degeneration and retinitis pigmentosa, childhood onset, signal abnormalities in cervical spinal cord	Sensory ataxia	[128, 129]

*HSP* hereditary spastic paraplegia, *LBSL* leukoencephalopathy with brainstem and spinal cord involvement and lactate elevation, *SPARCA1* spectrin-associated autosomal recessive cerebellar ataxia type 1, *SPAX* spastic ataxia, *SPG* spastic paraplegia

**Table 3:** Recessive disorders that may occasionally present with ataxia, but where ataxia is a secondary feature

Disorder	Gene	OMIM	Clinical features and imaging findings	Comment	References
Neuronal ceroid lipofuscinoses	*CLN5 CLN6*	256731 601780	Psychomotor retardation, visual failure, seizures, childhood to teenage onset, cerebellar and cerebral atrophy	Ataxia is a rare feature	[130, 131]
Sialic acid storage diseases (ISSD and Salla disease)	*SLC17A5*	604369 269920	Hypotonia, cerebellar ataxia and mental retardation, infantile to adult onset, cerebellar atrophy and demyelination	Complex syndrome	[132, 133]
Joubert syndrome	*AHI1*, *ARL13B*, *CC2D2A*, others	Many	Ataxia, hypotonia, neonatal breathing abnormalities, mental retardation, nephronophtisis, congenital onset, agenesis of the cerebellar vermis	Complex neonatal polygenic syndrome	[134, 135]
Hartnup disorder	*SLC6A19*	234500	Transient manifestations of pellagra, cerebellar ataxia and psychosis, amino aciduria, early onset	Metabolic disorder	[136]
Childhood ataxia with central nervous system hypomyelination/vanishing white matter disease	*elF2B*	603896	Cerebellar ataxia with spasticity. Rapid deterioration following head trauma or febrile illness, infantile to adult onset, diffusely abnormal cerebral white matter	Leukodystrophy	[137, 138]
L-2-Hydroxyglutaric aciduria	*L2HGDH*	236792	Psychomotor retardation, epilepsy, macrocephaly, cerebellar ataxia, infantile onset, subcortical leukoencephalopathy and cerebellar atrophy	Metabolic disorder	[139, 140]
GOSR2-linked progressive myoclonus epilepsy	*GOSR2*	614018	Ataxia, myoclonic epilepsy, raised creatine kinase, early childhood onset, variable cerebellar and cerebral atrophy	Epileptic disorder	[141]
Tremor-ataxia with central hypomyelination	*POLR3A*	607694	Tremor, cerebellar ataxia, cognitive regression, UMN signs, childhood onset, hypomyelination of deep white matter, cerebellar atrophy, thin corpus callosum	Leukodystrophy	[142]
Recessive Behr’s syndrome	*OPA1*	210000	Optic atrophy, ataxia, peripheral neuropathy, digestive symptoms, infantile or childhood onset, cerebellar atrophy	Optic atrophy	[143, 144]

*ISSD* infantile sialic acid storage disease

**Fig. 2:**
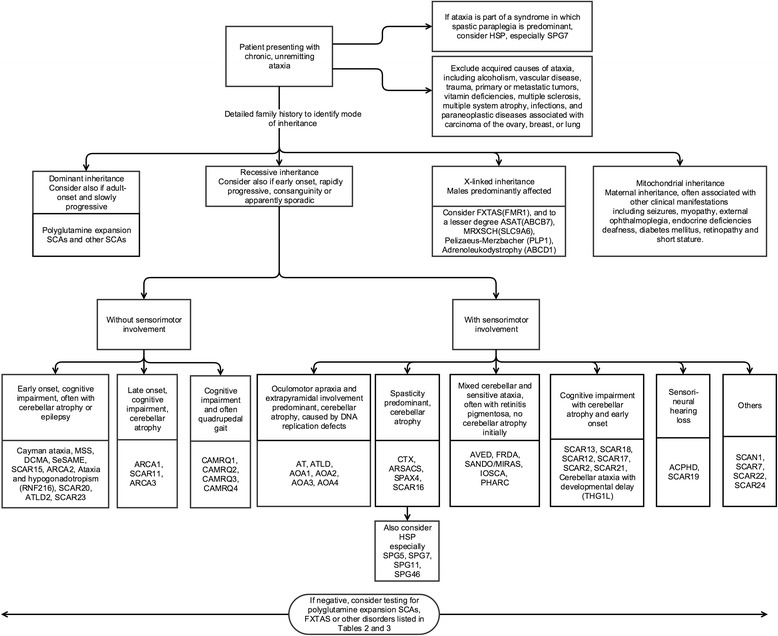
Clinical algorithm of autosomal recessive ataxias

The primary recessive ataxias were also organized in a clinical algorithm (Fig. 2) according to the presence of key clinical clues, which include the presence of sensorimotor involvement, cognitive impairment, spasticity, and oculomotor abnormalities.

Other disorders have been reported with ataxia, but the authors evaluated that these disorders did not need to be included in the differential diagnosis of recessive ataxias. However, clinicians may bear in mind that the following may have ataxia as an associated feature: Lafora disease (EPM2A, EPM2B), megalencephalic leukoencephalopathy with subcortical cysts (MLC1), COL18A1-linked ataxia epilepsy cognitive problems and visual problems, Perrault syndrome (HSD17B4), Zellweger-spectrum disorders (PEX2), Wolfram syndrome (WFS1), Canavan disease (ASPA), metachromatic leukodystrophy (ARSA), Galloway-Mowat syndrome (WDR73), and GLUT-1 deficiency (SCL2A1).

## Discussion

We present a new classification for the autosomal recessive ataxias. This classification should allow for better categorization of recessive disorders presenting with ataxia with a clear separation between the primary recessive ataxias and disorders that may present with ataxia as an associated feature but belong to other disease categories. We also provided a clinical algorithm as a tool for diagnostic, learning, and research purposes. This comprehensive classification will allow for improved genetic diagnosis by targeted next generation sequencing applications as the ability to detect DNA repeat expansion diseases is quickly becoming a reality with prospects of treatment in the future [11, 14, 15].

As compared to previously published reports on this subject [7, 8], we systematically reviewed the literature to evaluate the available evidence on the disease-associated genes in order to include all disorders presenting with a predominant cerebellar ataxia phenotype. The systematic review methodology with a structured data search and comprehensive evaluation of all references allowed for a complete evaluation of the literature regarding disorders presenting with ataxia to ensure that all potentially relevant disorders were included in this classification. Nevertheless, some methodological elements were not applicable to the task at hand. For example, two references were selected for each primary recessive ataxia, and articles that provided evidence for a separate genetic basis with a clinical corollary of ataxia were preferred. Therefore, some articles that provided only detailed clinical description were not included. Moreover, inclusion criteria were clearly defined but there remained a place for interpretation to determine if cerebellar ataxia was a core feature of the phenotype and if the genotype-phenotype association was convincing. Thus, the classification of individual disorders between the three groups, i.e. as a recessive ataxia, a complex disorder with predominant ataxia or a disorder where ataxia is a secondary feature, remains a subjective appreciation and is open for discussion by a dedicated task force in order to reach a consensus. Finally, the search strategy was designed to be as sensible as possible, but ataxia is a frequent symptom in neurology, and it is possible that other ataxia-associated disorders could be considered for inclusion.

Important challenges remain to be addressed. First, the nosology of recessive ataxias is still highly confusing. Contrary to the dominantly inherited spinocerebellar ataxias, no universal acronym was adopted in the field of recessive ataxias, such that disorders were named based on the author who first described them, on regions of high prevalence, or according to clinical presentation. In the last few years, the term spinocerebellar ataxia, autosomal recessive (SCAR) was used to designate novel recessive ataxias, but this nomenclature did not include the previously described and most frequent ataxias. Moreover, as SCAR assignation was based on locus discovery, some of the included SCARs do not correspond to an identified gene. The term SPAX has also been used to designate ataxias with a strong spasticity component, irrespectively of their mode of inheritance. Recently, the International Parkinson and Movement Disorder Society Task Force for Nomenclature of Genetic Movement Disorders recommended a nomenclature with a gene suffix in order to overcome the shortcomings of the numbered locus system, which include erroneously assigned loci, the mingling of causative and risk factor genes, unconfirmed causative associations, and inconsistent phenotypic correlations [16]. These concerns are justified, although numbered naming systems present definite advantages for ease of use and proper delineation of the field. The nomenclature of recessive ataxias should be discussed by a dedicated task force of international experts in order to develop a naming system that reflects the complexity of the recessive ataxia phenotypes while allowing convenient clinical use.

Finally, large phenotypic variability exists between patients from different families and even from a single family with the same mutated gene, depending on the type of mutation and on its location in the gene. Other factors that affect age at onset and clinical course probably include the presence of modifier genes and environmental exposures. Hence, one could argue that the paradigm of one gene-one disease presented here does not reflect all the phenotypic variability observed, and could as well be replaced by the concept of one patient-one disease as we identify new genetic and environmental prognostic features that characterise more precisely the age at onset, evolution, and response to treatment. Such developments are likely to modify our understanding of genetic disorders and of their classification.

## Conclusion

We present herein a classification of the autosomal recessive ataxias based on a systematic review of the literature. This work should serve as a framework for scientific discussion in order to bring together experts for the establishment of a much-needed consensus in this field.

## Additional file

Additional file 1: Search strategy for MEDLINE/PubMed. (DOCX 41 kb)
