# Supplementary material for: Systematic review of autosomal recessive ataxias and proposal for a classification
Source: Cerebellum Ataxias. 2017 Feb 23;4:3. doi: 10.1186/s40673-017-0061-y (PMC5324265; doi:10.1186/s40673-017-0061-y)
Supplement: Additional file 1: — Search strategy for MEDLINE/PubMed. (DOCX 41 kb) [file 40673_2017_61_MOESM1_ESM.docx]

**Additional file 1. Search strategy for MEDLINE/Pubmed**

1. recessive[TIAB] OR sporadic[TIAB]
2. ataxi*[TIAB] OR “spinocerebellar degenerations”[MeSH Major topic] OR “cerebellar ataxia”[MeSH Major topic]
3. gene[TIAB] OR genes[TIAB] OR locus[TIAB]
4. animals[MeSH] NOT humans[MeSH]

1 AND 2 AND 3 NOT 4
